# Hip Fractures: Therapy, Timing, and Complication Spectrum

**DOI:** 10.1111/os.12524

**Published:** 2019-09-30

**Authors:** Dominik Saul, Juliane Riekenberg, Jan C Ammon, Daniel B Hoffmann, Stephan Sehmisch

**Affiliations:** ^1^ Department of Trauma Surgery, Orthopaedics, and Plastic Surgery University Medical Center Göttingen Göttingen Germany

**Keywords:** Age traumatology, Complications, Femoral neck fractures, Hip fractures; Pertrochanteric fracture

## Abstract

**Objective:**

Investigation of the treatment of femur fractures and the type of femur fracture‐associated complications regarding timing of surgery and length of hospital stay.

**Methods:**

In this retrospective cohort study, a total of 358 hip fractures were evaluated retrospectively from 1 January 2008 until 31 December 2010 at a level I trauma center in Germany. Inclusion criteria was age >18 years and a proximal femur fracture. Both sexes were evaluated. Mean age was 75.5 years, most patients were female (63.7%). Intervention was the operative treatment of proximal femur fracture. Outcome parameters were time until surgery, complications, reoperations, mortality, and length of hospital stay.

**Results:**

Among the proximal femur fractures (*n* = 358), 46.6% were pertrochanteric, 11.2% subtrochanteric, and 42.2% femoral neck fractures. Operation upon hip fractures was managed regularly within 24 hours of injury (73%; mean for femoral neck: 28.3 hrs.; mean for pertrochanteric fractures: 21.4 hrs.; mean for subtrochanteric fractures: 19.5 hrs.). Delayed treatment, as well as implantation of hip total endoprosthesis (TEP), increased the overall length of hospital stay (15.4 *vs* 17.6 days; 18.1 *vs* 15.8 days). Accordingly, surgical procedures performed within 24 hours of injury resulted in a shorter hospital residence. Longest delay of operation was measured for hip fractures (28.3 hrs.).

In 351 patients, secondary injuries were detected in 94 individuals (26%), with fractures being the most common secondary injury (*n* = 40). We recorded postoperative complications of nonsurgical and surgical origin, and 33.6% of our patient cohort displayed complications. Complications were distributed among 118 patients. There was no significant difference in complications regarding the time of operation, with most nonsurgical and surgical complications appearing within 24 hours after operation (*n* = 110 *vs n* = 31). Nonsurgical complications, such as anemia (*n* = 49) and electrolyte imbalances (*n* = 30), were observed more frequently than surgical complications (*n* = 107 *vs n* = 34); however, these complications were reduced by delay in surgery (82.0% in 6–24 hrs. *vs* 74.2% in ≥24 hrs.). Anticoagulant therapy and age did not affect postoperative complications. The hospital mortality of patients was 6.2%. Follow‐up was restrained to ambulatory visits in the clinic.

**Conclusions:**

Surgical management of hip fractures performed within 24 hours of injury minimizes hospital stay. We did not detect significant differences in the spectrum or number of complications regarding delay of surgery. Surgical complications mainly occur with rapid primary care, and medical complications can be reduced by more intensive preparation of patient and operation procedures.

**Abbreviations**
ASA: American Society of Anesthesiologists
BMI: Body mass index
DHS: dynamic hip screw
HHA: hip hemi‐arthroplasty
PFN: proximal femoral nail
THP: total hip prosthesis

## Introduction

In Germany, hip fractures represent a major health burden, with a prevalence of approximately 135,000 cases per year^1^, ^2^, ^3^. Since demographic change is associated with a high proportion of elderly clientele, an increase of fractures of the femoral neck by at least 40% is estimated to occur until 2030^4^, ^5^, ^6^. The immense cost of €2–4 bn per year is mostly caused by age and prolonged release into the initial environment. One‐year mortality of hip fractures is alarmingly high at 20%–30%^7^, ^8^, ^9^.

Treatment of proximal femoral fracture is generally operative. An early operation leads to good results in femoral neck fractures due to reduced rates of head necrosis and 30‐day mortality^10^, ^11^, ^12^, ^13^. The ideal time for operation is controversially discussed, yet it is often shown to be less than 12 hours after the accident^14^, ^15^.

To prevent complications, such as ulcer, deep vein thrombosis, pulmonary embolism, and “surgical complications”, several organizations recommend an operation within 24–48 hours of injury for hip fractures^16^, ^17^, ^18^. A retrospective study was able to demonstrate a correlation between the number of days until operation and an elevated rate of total complications^19^. A Danish retrospective study showed that a delay of operation leads to increased 30‐ and 90‐day mortality after 12 and 24 hours respectively^15^, while an Italian study confirmed the 48‐hour limit for enhanced long‐term survival^20^.

The purpose of this study was to assess whether the time of operative treatment accounts for a particular spectrum of complications.

Additionally, we determined the average length of hospital stay and the underlying reasons.

Finally, we sought to investigate the treatment of femur fractures and which parameters lead to certain surgical complications, delayed operation, and reduced length of stay.

## Methods

### *Population and Inquiry Period*

All hip fractures (ICD‐10 S72.0, S72.1, S72.2) were retrospectively analyzed from 1 January 2008 until 31 December 2010 in a level I trauma center with a large sphere of referral in Germany. In this particular single center study, a consistent management throughout the 2 years with a simultaneously diverse operative repertory was performed.

A total of 351 patients with 358 proximal femoral fractures were included.

### *Inclusion criteria*

1. Age >18 years
2. Proximal femur fracture.

Operative treatment of proximal femur fracture, including DHS, PFN, THP, HHA, Screw, and others.

No treatment (conservative fracture management).

Mortality, periods between accident/admission and operation, length of hospital stay, complications.

Retrospective cohort study.

### *Measured Parameters*

#### *Patient Characteristics*

Age, sex, body mass index (BMI, kg/m^2^), American Society of Anesthesiologists (ASA)‐status^22^, concomitant injuries (other fractures, pneumonia, urinary tract infections), comorbidities (cardiological, nephrological, oncological etc.), medication and smoking status (yes/no) were collected at the entrance of hospital. Clinical significance of these parameters was whether they were linked to the time of operative care.

#### *Time of Operation*

The time of operation, the time between accident and operation, as well as the time between hospital admission and operating room were gathered for each patient retrospectively. A link between delay of operation and complication spectrum was sought.

#### *Complications*

Complications, reoperations, mortality and length of hospital stay (if deceased, the last day was considered) were assessed.

During the course of our study, nonsurgical and surgical complications were recorded. The complications were defined according to a selection of studies^23^, ^24^, ^25^, ^26^. Nonsurgical complications were defined as follows: anemia (hemoglobin <12.0 g/dL in women and <13.0 g/dL in men^27^), electrolyte imbalance^28^, symptomatic transitory psychotic syndrome^29^, cardiac or pulmonary complications [myocardial infarction^30^, pneumonia^31^], urinary tract infection^32^, renal dysfunction^33^, and thromboembolism^28^. Surgical complications included hematoma, mechanic malfunction [dislocations, cutting‐out, refracture], infections, necrosis, pseudarthrosis^34^, and healing in malposition according to the ICD‐10 in the patient's chart^35^.

#### *Operative Procedure*

The mechanism of injury, implant used (prosthesis, extra‐ or intramedullary implant), fracture classification (femoral head, pertrochanteric, subtrochanteric), postoperative course, rehabilitation, and living situation were also evaluated. We matched the applied implant and fracture type to the timing of operation and complications.

### *Statistics*

Data was collected using Microsoft Excel® (Microsoft Corporation, Redmond, WA, USA), and statistical analyses were performed using GraphPad PRISM 5.0 (GraphPad Software 5.04, San Diego, CA, USA). Comparisons of groups were conducted using the one‐way analysis of variance (ANOVA) and Tukey's *post hoc* test (α = 0.05). The level of significance used was *P* < 0.05.

## Results

### *Cohort Characteristics, Fractures, and Therapy*

The average age of our 351 patients was 75.5 years (74.2–77.1), and the cohort consisted of 36.2% male and 63.7% female patients. Seven patients had both‐sided proximal femoral fractures. Predominant ASA‐status was II‐III, and, on average, 2.7 accompanying illnesses were recorded per individual.

**Figure 1:**
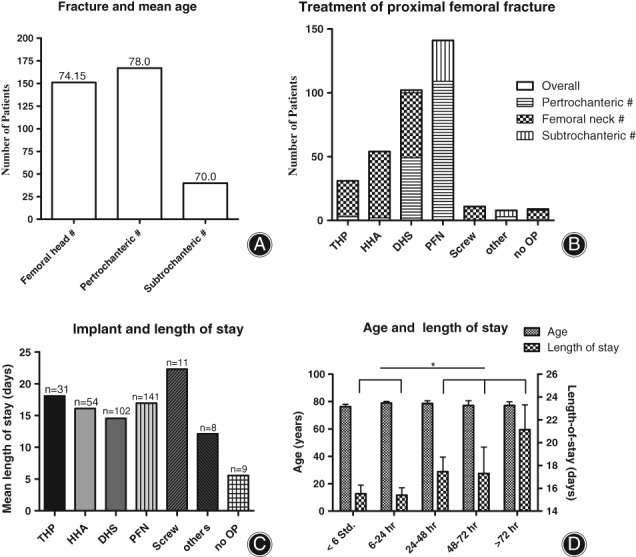
An early operative treatment reduced the duration of the hospital stay. (A) In this cohort, pertrochanteric femur fracture was the most common injury, followed by femoral neck fracture. (B) In hip fracture, PFN was used most frequently (40%), followed by DHS (28%). (C) Mean length of hospital stay for THP was 18.1 days (compared to all other with a mean of 15.8 days). (D) Mean age of patients operated on within the first 6 hours of injury was 74.1 years and did not differ significantly from the other groups. An early operation led to a length of stay of 15.5 days. The group of patients operated on either within or after 24 hours of injury differs significantly in length of stay (*t*‐test, *P* = 0.0374).

Throughout the 358 proximal femoral fractures, 167 were pertrochanteric (46.6%), while 151 affected the femoral neck (42.2%). Subtrochanteric fracture occurred in 40 cases (11.2%) (Fig. 1A). Seven patients sustained simultaneous both‐sided hip fractures.

In femoral neck fractures, dual head prosthesis (34.4%) and dynamic hip screw (DHS, 29.3%) were commonly utilized, while total hip prosthesis (THP) (18.5%) was used least. In pertrochanteric fracture, pertrochanteric femoral nails (PFN, 65.3%) and DHS (29.3%) were utilized. In subtrochanteric fractures, 80% were operated on by using PFN (Fig. 1B).

### *Periods between Accident/Admission and Operation, Length of Stay*

On average, 24 hours passed between a patient's admission and operation. Meanwhile, the time between a patient's accident and their hospital admission averaged 26.6 hours; however, with the exclusion of 10 outliers (125–672 hours) that average decreased to only 5.1 hours.

Patients with femoral neck fractures stayed 1 day less (15.4 days) compared to patients with pertrochanteric (16.6 days) or subtrochanteric (16.6 days) fractures. Between all types of implants used, no significant differences regarding length of stay were detected, with a median of 16.1 days in hospital (Fig. 1C).

Patients who underwent an operation within 6 hours of the accident stayed an average 15.5 days in the hospital, while operations within 6–24 hours and after 24 hours of injury were associated with stays of 15.4 and 17.6 days, respectively. Thus, surgeries performed after 1 day resulted in significantly longer stays (Fig. 1D).

**Figure 2:**
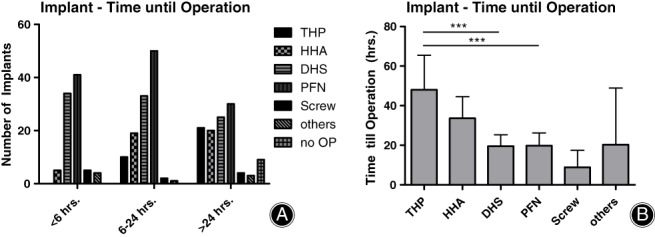
THP‐related procedures are associated with the longest delay until surgery. (A) While DHS and PFN were implanted regularly within 24 hours of injury, THP and HHA were regularly implanted >24 hours after the accident (THP *vs* PFN: *; HHA *vs* PFN *, PFN *vs* Screw: **, PFN *vs* others: **, PFN *vs* no OP: **; two‐way ANOVA, Bonferroni post‐test). (B) THP was implanted after an average of 48 hours, HHA after an average of 34 hours. Operation with DHS or PFN was significantly faster compared to THP (*P* = 0.0001 [***] and 0.0009 [***] respectively, One‐way ANOVA, Tukey's *post hoc* test 5% level of significance).

The highest delay in operation was seen in relation to the usage of THP, while hip hemiarthroplasty (HHA) was regularly implanted within the first 6 hours of injury (Fig. 2A, B).

### *ASA‐Status, Anticoagulants, Timing of Operation*

**Figure 3:**
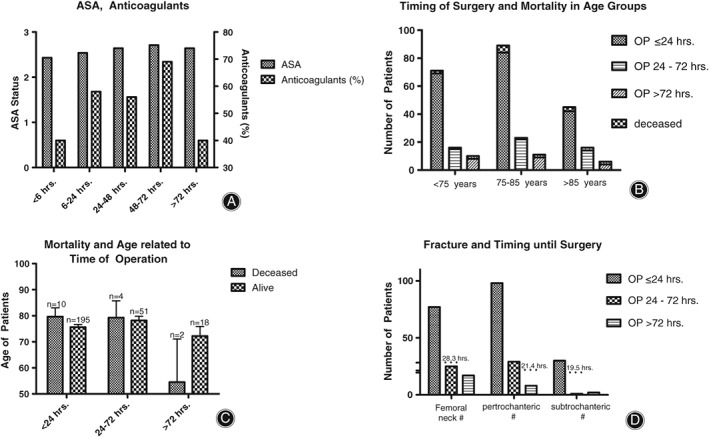
Based on ASA status and mortality, age did not affect the clinical outcome. (A) ASA‐status did not differ significantly among the groups. Anticoagulants were administered to more patients the longer their operation was delayed, while in the >72 hours group, only 40% were treated. Immediate operation was significantly rarer when anticoagulants were taken (Chi‐Square test with 5% level of significance, *P* = 0.0152). (B) Over all age groups, operation was mostly performed within 24 hours of injury (*P* = 0.0058 [**], two‐way ANOVA with 5% level of significance). The deceased patients were distributed equally among the age groups and there were no differences in mortality between the groups (*P* = 0.7486, one‐way ANOVA with Tukey's *post hoc* test and 5% level of significance). (C) The deceased patients that were operated on within 24 hours of injury were slightly older than those operated on after >72 hours. In the latter group, the survivors were older than the deceased patients, although this finding was not significant (*P* = 0.65; *t*‐test, unpaired, 5%‐level of significance). (D) Operation on femoral neck fractures took place an average of 28.3 hours after the time of injury, although most patients were operated within 24 hours of injury. Pertrochanteric fractures were regularly operated on within 24 hours of injury, with a mean time until operation of 21.4 hours.

Subgroup analysis showed that ASA‐status did not differ significantly among the groups (Fig. 3A), while anticoagulants were used significantly less frequently in patients operated on within <6 hours of injury (Fig. 3A).

Operation upon hip fractures was managed regularly within 24 hours of injury (Fig. 3B). Interestingly, patients who were operated on after 72 hours and survived were older than patients who were operated on after 72 hours and did not survive (Fig. 3C). Delay of operation was the longest for hip fractures, with 28.3 hours until operational treatment, and the shortest for pertrochanteric fractures (21.4 hours, Fig. 3D).

### *Secondary Injuries, Spectrum of Complications*

**Figure 4:**
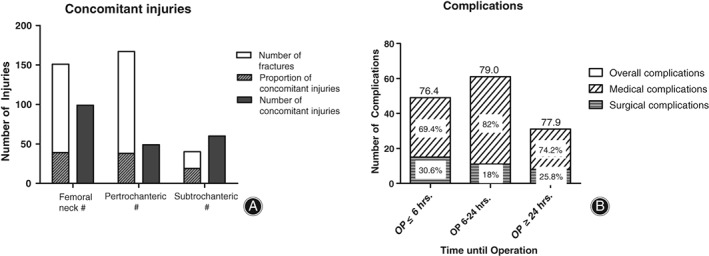
Hematoma and anemia were the most common complications. (A) Concomitant injuries occurred in 25.8% of femoral neck fractures, with 0.67 injuries on average per patient. Pertrochanteric fractures had 24.8% concomitant injuries, with 0.33 injuries per patient. Subtrochanteric fractures had the most concomitant injuries (46.5%), with 1.5 per patient on average. (B) Most complications were measured if the operation was performed within 6 to 24 hours of injury, while most surgical problems occurred within 6 hours of surgery. Age did not differ among the groups (*P* = 0.4195, one‐way ANOVA, Tukey's *post hoc* test, 5%‐level of significance).

In 351 patients, secondary injuries were detected in 94 individuals (26%), and the most common secondary injuries were fractures (Fig. 4A). We recorded postoperative complications of nonsurgical and surgical origin, and 33.6% of our patient cohort displayed complications. Complications were distributed among 118 patients, whereas surgical complications were most common in patients operated on within 6 hours of injury (Fig. 4B).

**Figure 5:**
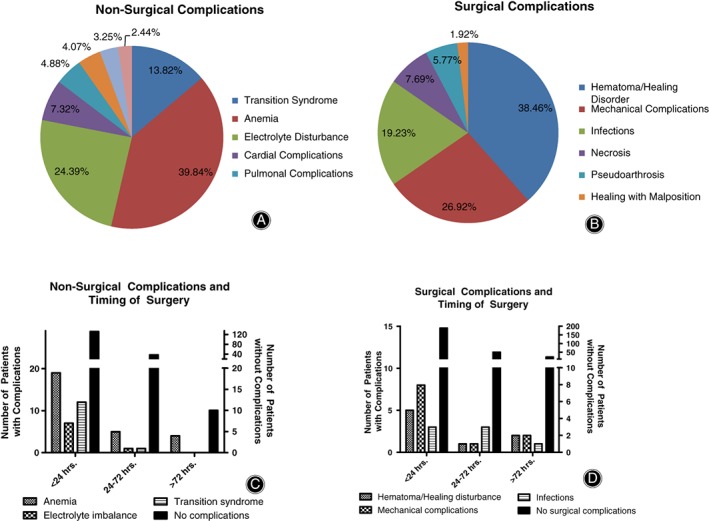
Non‐Surgical and surgical complications show a predominance if operation was performed within the first 24 hours of injury. (A) The relative frequency of nonsurgical complications shows anemia (37.7%), electrolyte imbalances and transition syndrome as the most common complications, while (B) frequency of surgical complications demonstrates that hematoma/healing disturbances were most common (38.5%). (C) When operations were performed within 24 hours of injury, anemia and transition syndrome were most common in patients; however, anemia became rare with a longer period before operation (without leading to significant differences, one‐way ANOVA with Tukey's *post hoc* test). (D) In most patients, no surgical complications were measured. When operations were performed within 24 hours, mechanical complications and hematoma/healing disturbances were most likely to occur in patients (no significant differences in one‐way ANOVA with Tukey's *post hoc* test).

Nonsurgical complications included anemia, electrolyte disturbances and transitory psychotic syndrome (Fig. 5A). Among surgical complications, mainly hematoma and mechanical complications such as dislocations, cutting‐out, or refracture were detected (Fig. 5B).

There was no significant difference in complications regarding the time of operation, with most nonsurgical and surgical complications appearing within 24 hours after operation (Fig. 5C, D). Most complications occurred when using the PFN, while relative reflection depicts this implant as most secure (Table 1).

**Table 1:** Relative complications divided into nonsurgical and surgical show THP as the implant with the lowest risk of surgical adverse events. THP leads to complications most commonly, while PFN und DHS – relatively considered – had the fewest complications. Regarding just surgical complications, THP was the lowest‐risk implant, followed by HHA and PFN

	THP	HHA	DHS	PFN	others
Overall complications (Proportional to operations,%)	61.23	55.54	48.03	46.09	84.21
*Non‐Surgical complications (%)*	54.81	44.44	31.38	32.62	47.37
*Surgical complications (%)*	6.46	11.11	16.67	13.48	36.84
*Number of patients*	31	54	102	141	19

### *Mortality*

Twenty‐two patients died during their hospital stay (6.2%). These patients were older than the collective, although not significantly. Time until operation did not differ from the surviving collective.

## Discussion

Studies addressing hip fractures report cohort characteristics comparable to those of our study^36^, ^37^, ^38^, ^39^, with a greater proportion of women^40^, ^41^ and a similar proportion of comorbidities^42^, ^43^.

### *Length of Hospital Stay*

Regarding hip fractures, several studies reported an average stay in hospital of between 13 and 20 days when PFNs were implanted^44^, ^45^, ^46^, which is similar to our finding of 16.1 days. The longer hospital stay resulting from the usage of THP that we measured was confirmed by a German study, with 21.3 days of residence for prosthesis^47^. One possible explanation for the prolonged stay could be the longer time until operation. Shorter treatment times for patients with femoral neck fractures may be due to the earlier release of this cohort into a nursing home or short‐term care. The longer time until release shown for subtrochanteric fractures may underlie the greater force of impact for this kind of fracture, resulting in more concomitant injuries.

The minimization of time from patient admission to operation can reduce complications and shorten the length of their stay^13^. Length of hospitalization has tremendous financial implications since DRG‐implementation^48^, ^49^, ^50^. In this study, time until admission is significantly longer after exceeding the 24‐hour operation limit (17.6 *vs* 15.5 days). This time limit is confirmed by several studies^51^, ^52^, ^53^. Neither ASA‐status nor age of patients is significantly correlated to the timing of operation. Age of surviving patients is indeed correlated to the time of operation. Additionally, the intake of anticoagulants is correlated to the timing of operation, as we and several other studies were able to demonstrate^54^, ^55^, ^56^. An early operation may reduce mortality^7^, ^57^, which could not be proven by this study.

### *Spectrum of Complications*

In this population, 33% of patients had complications, with 11.5% of them being surgical. Comparable studies show rates of 12.5%–40%^46^, ^58^, ^59^, ^60^, ^61^. Regarding nonsurgical complications, anemia and electrolyte imbalances were predominant, which confirms the rate of 6%–8% found in other studies^43^, ^62^.

We strictly separated hematoma from postoperative anemia in our population. While the latter accounted for the greatest part of nonsurgical complications in this study, other authors report up to 86% anemia after operations on hip fracture and tend to not list this as a complication^63^. In a Spanish analysis, a 24%–44% rate of anemia was described, which is close to our rate.

The time‐of‐operation and implant‐used variables show that nonsurgical complications (anemia and transitory psychotic syndrome) were mostly seen when the operation was performed in the first 24 hours; a longer preparation tends to reduce medical complications. An Israeli study shows that the positive effect of a fast operative procedure on mortality cannot be maintained for longer than 6 months^64^. Comorbidities especially seem to cause prolonged preparation for operative procedures^65^, ^66^, ^67^.

Surgical complications – especially hematoma and healing disorder with a frequency of 5.6% – were reported in this study, particularly after DHS and PFN, which is in accordance with the literature^61^, ^68^. A correlation between the intake of anticoagulants and either hematoma or risk of infection was not seen in this analysis. Infections were a rare complication (2.8%) and comparable to those rates previously reported (1.5%–3.8%^43^, ^69^, ^70^). A “collapse of osteosynthesis,” which the literature suggests can be expected in 3.4%–7.7% of patients^39^, ^71^, was seen in 3.9% of our study patients.

Femoral head necrosis has been detected in 1.1% of all fractures in the study; however, long‐term results remain to be collected, and the expected 11.8%^34^ suggests that the follow‐up was too short.

The fact that most surgical complications appeared within the first 24 hours after the operation could be due to the emergency aspect or lack of expertise of the primary operating surgeon, although the latter should not have any influence^72^, ^73^, ^74^. Referring to the implant used, no significant differences between DHS, THP, or PFN in surgical or nonsurgical complications were seen, which is confirmed by other surveys^75^.

Revisions were necessary in 11.5% of our patients because of surgical factors. Since similar studies report rates of 5.5 to 53%^10^, ^46^, ^76^, grading seems complicated.

Smektala *et al*.^13^ proclaimed that an early operation leads to reduced complications and higher survival rate. While an early operation seems to reduce the rate of complications^77^, ^78^, and a reduction of 36 to 24 hours before the operation raises the rate of survival^7^, we aimed to achieve an early treatment. Within 24 hours of injury, 58% of our patients were surgically supplied, which is close to comparable studies^43^, ^46^, ^61^. Primary THP was operated on after an average of 48.0 hours, while osteosynthesis was performed after a median of 15.4 hours, thereby demonstrating a significant difference. Clientele who are operated on within 6 hours of injury are marginally younger and have a lower ASA‐status than patients treated after the 6 hour period, which can be confirmed by the literature^12^, ^61^, ^79^. Patients older than 85 years are less frequently operated on within 24 hours of injury than are younger patients. A preoperative stabilization partly seems to provide survival benefits to patients^80^, which supports their supply in specialized age‐traumatology centers^81^, ^82^, ^83^.

### *Mortality*

Hip fractures are associated with mortality rates of 5.4%–14.3% in the literature^37^, ^39^, ^43^; our rate of 6.2% lies within this range. The influence of age or sex could not be confirmed in our study^84^, ^85^, ^86^. Older patients seem to profit from a more intensive preparation for operation, which supports a collaboration between trauma surgery and geriatrics^81^, ^82^, ^87^; however, the operation threshold of 24 hours needs to be maintained and could be recently confirmed^88^, ^89^. A patient cutoff of 85 years of age is difficult to be determined, but in a recently published study with 2,000 patients an age of >82 years resulted in higher two‐year mortality^89^. The data seems to support a difference between the older and younger patients, but conducting a thorough physical examination, basic lab tests, and collecting the general health history and activity status of the patient needs to be the basis of making the decision about when to operate.

### *Limitations*

The present study has been conducted retrospectively for 358 fractures. Incomplete patient records reduce the informative value of the inquiry. Follow‐up is restrained to ambulatory visits in the clinic and is therefore insufficient to detect long‐term complications, consolidation status, or long‐term results. An important bias could be that the patients operated on early were probably those in a better general condition, which could falsify the conclusion of these having better outcome parameters. Preoperative hemoglobin was not detected, and the definition of “anemia” is simply based on the postoperative hemoglobin value.

Regression analyses for potential confounders have not been fully performed, which is why some of the reported results may be due to confounding.

### *Conclusion*

For hip fractures, surgical and nonsurgical complications arise in 33% of patients, with the former occurring more rarely at 11.5%. For the oldest patients, where comorbidity could an interfering factor, intensive preparation for operation seems to be beneficial, while delaying the operations longer than 24 hours increases the patient's length of stay in the hospital.

## Declarations

### *Ethics Approval and Consent to Participate*

Approval to analyze patient data was received on 6 January 2014 by the institutional review board (IRB) of the University Medical Center Göttingen (Ethikkommission der Universitätsmedizin Göttingen, Von‐Siebold‐Str.3, 37075 Göttingen). The patients' consent to participate was recorded in writing.

### *Consent for Publication*

Not applicable.

### *Availability of Data and Material*

The datasets used and/or analyzed during the current study are available from the corresponding author on reasonable request.

### *Competing Interests*

The authors declare that they have no competing interests.

### *Funding*

Not applicable.

### *Authors' Contributions*

JR collected and interpreted the data. DS and JR analyzed and evaluated the data. DS wrote the first version of the manuscript. JR, JCA, DBH, and SS re‐wrote specific sections of the manuscript. All authors read and approved the final manuscript.
